# Use of electronic patient diaries supports diagnosis of food allergy and diet management

**DOI:** 10.1186/2045-7022-1-S1-O38

**Published:** 2011-08-12

**Authors:** Andreas Arens-Volland, Frank Feidert, Ralf Herbst, Ralph Mösges, Norbert Rösch

**Affiliations:** 1 Public Research Centre Henri Tudor, SANTEC, Luxembourg, Luxembourg; 2 Centre Hospitalier de Luxembourg, ORL-Eich, Luxembourg, Luxembourg; 3 University Hospital of Cologne, IMSIE, Cologne, Germany

## Background

Patient diaries have the potential to support food allergy diagnosis. The time relationship between the consumed product and the experienced symptoms is of high value for diagnostics as well as for therapy control. The combination of a barcode reading handheld device and a dedicated electronic patient record is a unique method to support the diagnostic process.

## Methods

A Smartphone based Personal Allergy Assistant (PAA) allows patients to keep an electronic patient diary by scanning the barcode of the consumed food products. A catalogue of predefined symptoms as well as their medically justifiable time window during which a symptom can manifest itself after the ingestion has been defined. An Electronic Patient Record for Allergies (EPRA) stores results from patient investigations with allergy specific medical knowledge, provided by allergy experts (e.g. cross allergies, seasonal effects, pollen associated FA). Data on the PAA is regularly synchronised with the electronic patient record and the food database. Diaries are evaluated by emphasising the association between food intake and occurred symptoms in order to calculate the relative risk (RR) of a specific food product or nutrition.

## Results

The PAA provides patients with an easy to use device to create high quality diaries for food intake and symptoms by scanning the food package's EAN barcode. It sets automatic timestamps to reduce potential data tampering and increase the data quality and allows recording of food intakes from non-packaged food (e.g. fruits, vegetables or fast food) as well as pharmaceuticals. For diagnostic purpose, the diary is regularly transmitted to the allergist's electronic patient record. Diaries are evaluated automatically providing measurement categories that are commonly used in medicine. To support the individual diet management, the PAA gives a warning before the consumption of allergenic food.

## Conclusions

During a pilot study with 35 participants the feasibility and patient acceptance has been proven. Information and Communication Technology has the potential to simplify the interdisciplinary exchange of patient information between patients and medical experts.

